# Population-based neuropathological studies of dementia: design, methods and areas of investigation – a systematic review

**DOI:** 10.1186/1471-2377-6-2

**Published:** 2006-01-09

**Authors:** Julia Zaccai, Paul Ince, Carol Brayne

**Affiliations:** 1 Department of Public Health and Primary Care, University of Cambridge, Robinson Way, Cambridge CB2 2SR, UK; 2 Academic Unit of Neuropathology, University of Sheffield, 'E' Floor, Royal Hallamshire Hospital, Glossop Road, Sheffield S10 2JF, UK; 3 Department of Public Health and Primary Care, University of Cambridge, Robinson Way, Cambridge CB2 2SR, UK

## Abstract

**Background:**

Prospective population-based neuropathological studies have a special place in dementia research which is under emphasised.

**Methods:**

A systematic review of the methods of population-based neuropathological studies of dementia was carried out. These studies were assessed in relation to their representativeness of underlying populations and the clinical, neuropsychological and neuropathological approaches adopted.

**Results:**

Six studies were found to be true population-based neuropathological studies of dementia in the older people: the Hisayama study (Japan); Vantaa 85+ study (Finland); CC75C study (Cambridge, UK); CFAS (multicentre, UK); Cache County study (Utah, USA); HAAS (Hawaï, USA). These differ in the core characteristics of their populations. The studies used standardised neuropathological methods which facilitate analyses on: clinicopathological associations and confirmation of diagnosis, assessing the validity of hierarchical models of neuropathological lesion burden; investigating the associations between neuropathological burden and risk factors including genetic factors. Examples of findings are given although there is too little overlap in the areas investigated amongst these studies to form the basis of a systematic review of the results.

**Conclusion:**

Clinicopathological studies based on true population samples can provide unique insights in dementia. Individually they are limited in power and scope; together they represent a powerful source to translate findings from laboratory to populations.

## Background

Within the next two decades unprecedented numbers of people will be entering the age range at which incidence of dementing diseases is highest. The EURODEM Prevalence Research Group estimates that 6% of people over the age of 65 will suffer from dementia, with 30% in the over 80s [1]. The economic cost of Alzheimer's Disease (AD) is already higher than that of heart disease and cancer combined [2]. The high dependency associated with dementia, with its costs and consequences for society, gives research into the aetiology and pathogenesis of late life dementia major priority.

**Figure 1:**
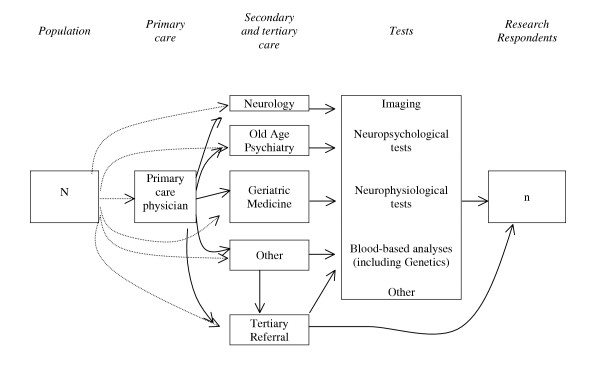
Pathways through which individuals may be filtered before becoming potential research respondents. Note: In systems such as in the UK, referral from secondary and tertiary services is almost always through primary care. This is not the case in all health care systems. N = true population. n = study

Dementia research based on human brain tissues ranges from observation of the pathology of selected individuals to investigate the molecular mechanisms of dementia to population-based studies to understand who, where and why people develop dementia [3-7]. A substantial contribution to our understanding of the pathology, neurochemistry, molecular pathology and genetics of different types of dementias has arisen from the observation on the brains of individuals who have been demented during life. These studies have been key to recent advances in understanding dementing disorders. All clinicopathological studies can investigate the performance of in life diagnostic methods to after life brain appearances but very few studies have combined this detailed molecular approach to understanding the neuropathological findings in a population context. Most pathological studies in the elderly have drawn on cases from nursing homes, acute medical units, hospital patients, volunteer cohorts or ordinary post-mortem series [8,9]. However, it is known that selection bias resulting from referral of patients from primary to secondary and on to tertiary care centres can affect profoundly the results of clinical or epidemiological studies [10]. This is because referral is influenced by more than the severity of the disorder itself and has much to do with the way that communities contain and deal with aberrant behaviour [11]. Referral may differ according to burden of symptoms, access to care, popularity of disorders specialisation and institutions (e.g. the 'Berkson paradox' [10]). Furthermore specialised patient research groups derived from referrals typically use stringent selection criteria so that patients are usually selected to have fewer co-morbid conditions. Potential referral biases have rarely been investigated systematically in studies of pathology in dementia cohorts. Differences in sociodemographic characteristics of three groups of patients with AD from different sources in the US have been reported as significant. The patients being compared were population-based patients, patients referred from a near distance (primary or secondary care), and patients referred from a far distance (tertiary care) [12]. Such biases in sociodemographic variables have important implications for research because some of these variables have been implicated as risk factors for AD. One of these is age at onset of symptoms, which can be a marker of the severity, the genetic nature, and the clinical course of AD. The discrepancy between the general population and samples of research respondents in cohort studies based on medical access is shown in Figure 1. It is usually not possible to determine whether the findings on a selected research group can be generalised to the whole population, and it is likely that mostly they are not. This also applies to all volunteer cohorts. For example, the Nun study respondents are members of the School Sisters of Notre Dame religious congregation. These participants, who have chosen to be a member of a group rather than being born into it, have been in a controlled geographical and social environment with little heterogeneity of exposure of potential risk factors since puberty. This has some advantages in investigating specific hypotheses but it is difficult to estimate the effects of this selection on the generalisability of any findings to the population as a whole. This means claims regarding the possible effect size of risk factors on the importance of any particular pathology are often based mostly on potentially biased findings in highly selected populations. The proportions of various types of dementing disorders could be artificially skewed by the participation of a non-representative sample of the total demented population. For extrapolation of results to the population to be valid, research must be conducted on a true population sample, or on groups with well characterised biases. Working with truly population-based studies is the best way to minimize misinterpretation and bias.

Beyond the pathological confirmation of diagnosis, and the inherent generalisability of the data, working with population-based neuropathological studies allows a range of further investigations (along with cohort studies). These include identification of brain lesions that relate best to cognitive decline, and the examination of links between pathologies and potential risk factors. This approach highlights the significance of brain lesions as substrates for the decline noted in normal ageing in comparison to the demented [11]. Because population cohorts naturally represent spectra of disease, it is possible to test hypothetical semi-quantitative thresholds definable in terms of a staging system [13,14] that might discriminate clinical dementia and illuminate critical steps in the neurobiological progression of cognitive decline through states such as Mild Cognitive Impairment. Thus identifying neuropathological markers relevant to cognitive function both helps in the diagnostic process and the understanding of the molecular and clinical aetiology of dementias and ultimately results in the development of better treatments.

Prospective population-based neuropathological studies allow findings of clinicopathological cohort studies to have a special place in dementia research which is under emphasised. This paper reports a systematic literature review of the methods of on-going population-based neuropathological studies of old age dementia, assesses the representativeness of the study populations included in each, and reviews their neuropathological methods. Examples of findings from population-based studies will be discussed to illustrate the range of areas investigated.

## Methods

### Literature data sources and search strategy

A systematic review is the application of strategies that limit bias in the assembly, critical appraisal and synthesis of all relevant studies on a specific topic [115]. Systematic reviews focus on peer-reviewed publications about a specific health problem and use vigorous, standardised methods for selecting and assessing each article. A search was performed in July 2004 using the entire Medline, PubMed, Embase and Web of Science databases without using any language restrictions. The following key words were used to perform multiple searches: dementia, neuropathol*, autopsy, population, community. The search was not straightforward in that it was difficult to pinpoint an adequate MeSH word in PubMed or use the explode options in other databases to select for truly population-based studies. To solve this problem keywords cited with relevant articles already retrieved were scoped and used in the search strategy. However, this approach did not yield additional target publications. A preliminary list of 4,048 papers were traced and a first selection was made based on their title and abstracts. These 4,048 papers do not represent 4,000+ different cohorts but all the publications from a smaller number of cohorts. Key authors were contacted and key studies were also followed in order to trace descriptions of original study designs. The search was discontinued when no new studies were being found. Data within articles directly reporting population-based neuropathological studies of dementia in the elderly were extracted using a data collection proforma designed specifically for this review.

### Study selection

The aim of the literature search was to gather information on all studies that are population-based neuropathological studies of dementia. These are studies where a general population defined by geographical boundaries is the sampling frame [115]. Respondents must be recruited from all sub-groups of the population, whatever the social background, residential status (community or institutional) or health status. Studies were included if they did not have selection criteria for the respondents related to 'caseness' or potential 'caseness', and if they sought post-mortem examination of the brains of respondents across all cognitive states.

## Results

### List of studies and study populations meeting inclusion criteria

Six studies were found to be true population-based neuropathological studies on old age dementia. They are briefly described by date of recruitment of baseline population. Reference lists for each study are not exhaustive.

The Hisayama study is a prospective cohort study, which has been carried out since 1961 in a Japanese subrural community in Hisayama, Japan. The initial aim was to explore the epidemiology of cerebrovascular diseases. Starting in 1985, the prevalence of dementia was investigated among residents aged 65 and over. The study recruited a series of three cohorts (in 1985, 1992 and 1999) and in 1992 it had a huge response rate at baseline with 99.7% (= 1436) of the eligible population enrolled. Between 1985 and 1992, 82% (= 176) of the participants who died underwent brain examination at autopsy; between 1998 and 2001, the autopsy rate was 70.5% [15-19].

The Cambridge City over 75 Cohort Study (CC75C) is a long-term British follow-up study of a population sample of around 2,600 people aged 75 and above, also begun in 1985 [143]. The study started as the Hughes Hall Project for Later life (dementia prevalence phase) then became the Cambridge Project for Later Life (first incidence phase), before being renamed CC75C. The original study targeted all people who were registered with selected group general practices in Cambridge (sampling based on availability of universal health care free at the point of delivery in the UK), including those in institutions, and achieved a 92% response rate at baseline [20-26].

The Vantaa 85+ Study is based on an unselected prospective population-based sample of all individuals aged 85 or over living in the city of Vantaa, in Southern Finland, on April 1, 1991. There was a 92% response rate with 553 subjects taking part in the study. The Vantaa 85+ has the second highest proportion of autopsies of all the selected studies with 55% of the initial population having donated their brain by 2002 [27-33].

The Medical Research Council Cognitive Function and Ageing Study (CFAS) is based on six centres in England and Wales (Cambridgeshire, Gwynedd, Newcastle upon Tyne, Nottingham, Oxford and Liverpool). The study population was a random sample from registers of general practices, of around 2,500 people for the first five centres and 5,222 people in Liverpool. The response rates were 82% for the five first centres, and 87% in Liverpool so that there were 18,131 respondents at baseline. The only selection criterion for initial screening was age, respondents being 65 years old and over at baseline (1991–93) [34-44].

The Honolulu-Asia Aging Study's (HAAS) source population are survivors recruited in 1991 from the cohort of the Japanese-American men who were examined as part of the Honolulu Heart Program from 1965 to 1971 and re-examined in late age. The original cohort of 8,006 respondents included all men born between 1900 and 1919 and living on the island of Oahu and represented 65% of the target population. At the 1991 baseline there was a 79.8% response rate with 3,734 subjects taking part in the study of cognition (30% of the original cohort contacted in 1965). One of the major objectives of HAAS was to compare rates of dementia in a cohort of Japanese-American men in Hawaii with rates of Japanese living in Japan and the United States mainland [45-63].

The Cache County Study of Aging and Memory's (Cache County study) source population are residents (including those living in institutions) of Cache County, Utah (USA), who were aged 65 years or older on January 1, 1995. This population has an exceptional longevity, especially for men whose conditional life expectancy at age 65 is the highest in the United States and exceeds national averages by almost 10 years. There was an 89.7% response rate with 5,092 subjects taking part in the study at baseline [64-79].

Core information for these six studies can be found in (Table 1). Clinical and neuropathological standards and tests used by the studies are in (Table 2).

### Examples of excluded studies

Additional File 1 shows examples of studies identified during the search but which did not meet the inclusion criterion in that respondents recruited to the neuropathological component should include all subgroups of the relevant local population. All the non-selected studies were based on people referred to various clinics, people living only in institutions or only in the community, people signed up on a non-universal health care system or volunteers. The geographical boundary was not a criterion that excluded studies in itself but chosen because it offers the clearest way of avoiding selection bias which may be related to dementia status. While many excellent population-based cohorts exist (PAQUID, ILSA, The Rotterdam Elderly Study, etc), few have collected neuropathological data [3-5,80-89].

### Hypotheses tested in the six existing population-based neuropathological studies

Additional File 2 shows the scope of investigations presented in publications from the six retained studies. A summary of the findings for a selected number of papers is included.

The key areas from these studies include:

(1) Clinicopathological correlation and use of postmortem-confirmed diagnosis (most frequent type of published work);

(2) Assessing standards and staging models;

(3) Association between neuropathologies and risk factors;

(4) Genetic investigation in relation to autopsy-confirmed diagnosis or specific neuropathologies.

## Discussion

### Characteristics of the cohorts

All six studies chose their source populations according to geographical area and age. The Hisayama study, CFAS and the Cache County study selected for a population aged over 65, while HAAS selected for over 72, and CC75C for over 75. The Vantaa 85+ study included all individuals aged 85 and over thus representing the very elderly. Interpretation of findings must take these design strategies into account. For example, in a study of people surviving to 85+ years differential survival rates may affect genetic findings such as true homozygous for Apoe E4 individuals because they are at increased risk for premature death from cardiovascular disease and similar causes, and therefore likely to be underrepresented in populations of older individuals [90]. Studying a very elderly population may introduce new sources of bias but also provide new insights.

By definition all six studies sought community-dwelling and institutionalised respondents. This requirement to include institutionalised persons in a representative community sample in dementia studies has been challenged. Based on a meta-analysis covering prevalence studies from 1945 to 1985, Jorm and colleagues found no effect on the overall prevalence rates of dementia [91]. They argued that institutionalised individuals represent only a small proportion of the elderly population and thus effects are marginal. However, there are secular changes in institutional rates and variations across countries and cultures [92]. In a German study of people over 75 (LEILA75+) it was found that institutionalised individuals were seven times more likely to be demented than community-dwelling individuals, but they were also older, more often single and less often married [93]. In the CFAS population, 62% of those living in institutions were demented and represented 34% of those with dementia in the whole study cohort [94].

Each study population has characteristics which influence how generalisable the findings are, but which may also relate to the rationale behind selecting the study area. The Cache County study sample was chosen because it was not typical of the present USA population as a whole. A great majority of the respondents did not smoke or drink alcohol and 91% were members of the Church of Jesus Christ of Latter-Day Saints – factors which may contribute to the average longevity among men in the study that exceeds the USA national average by almost 10 years. HAAS is also restrictive in the sense that it only includes men, who do not represent the majority of dementia sufferers in the population. It was created out of several cohorts with a different initial purpose, which was the investigation of the effect of migration on vascular risk. It could be argued that these two studies are more akin to the selected cohort studies than to the true population-based studies in that there may be limited generalisability from their findings. CFAS is the only multicentre study mixing populations from both rural and urban settings but has little ethnic variation.

To date these studies have not presented their findings with any attempt to weight back to the population. For now, study size, initial response rate, and attrition are important factors that determine whether a study using an autopsy endpoint is truly population-based. Large study populations are needed for adequate statistical power to detect relationships between variables, such as the effect of specific gene polymorphisms. Initial response rates and follow-up rates need to be high, and maintained, to retain the representativeness of the group. All six studies showed high initial response rates. These range from 79.8% (HAAS, sampled from the 65% follow-up cohort studied from 1965–1971), to 92% (Vantaa 85+ and CC75C) and a remarkable 99.7% for the Hisayama study in the 1992-screening wave. Attrition varied across the studies. Due to the greater age of the baseline Vantaa 85+ respondents had a higher death rate between each follow-up compared to the other studies. Approximately one-half of the subjects had died by the 3-year follow-up but 97.3% of survivors were re-examined [95]. In CC75C, the two-year incidence wave achieved 67% follow-up of all respondents, and an 81% follow-up of all survivors [23]. Similar reporting of attrition is made in CFAS, HAAS and the Cache County study publications.

The problem of non-response can be especially important for the population study of cognitive status in the elderly because cognitive compromise is a predictor of non-response. Non-response in the Cache County study was related to lower levels of cognitive functioning, as measured by the MMSE, than initial responders [74]. They also reported that the sample under-represented females, younger individuals and people from other ethnic or religious backgrounds than members of the Church of Jesus Christ of Latter-Day Saints. The CC75C study also reported a lower baseline MMSE score (-1.17) in those who did not participate at follow-up, although 15% of these non-responders were initially interviewed but did not complete the assessment [36]. It is likely that these MMSE scores were disproportionately low at the interview and so the effect of MMSE on not being interviewed at all will not be as high as -1.17 [96]. In CFAS, looking at longitudinal attrition, predictors for drop out due to death were being older, male, having impaired activities of daily living, poor self-perceived health, poor cognitive ability and smoking. Similarly individuals who refused were more likely to have poor cognitive ability but had less years in full-time education and were more often living in their own home though less likely to be living alone [97]. Further investigation into the characteristics of those lost to follow-up after initial enrolment was not reported by the other selected studies. This also includes investigating those lost to follow-up because of death. The Vantaa 85+ study and CFAS report that death certificates were checked to identify new dementia cases among those who were not demented in the last interview. This strategy is undermined because the recording of dementia on death certificates is notoriously unreliable [98-100]. Other longitudinal studies of ageing and dementia have also suggested that mental status scores predict attrition [101] so that the influence of non-responders on the findings should not be minimised. In spite of this, none of the six studies could be excluded according to this criterion. These are all large population studies dealing with the older population where in any case high attrition is expected and where ethical approval usually does not allow approach of those who decline future participation.

### How representative were the autopsy samples compared to all deaths in the initial cohorts?

In volunteer cohorts such as the Nun Study, very high rates of autopsy amongst the deaths are achieved. How do population-based studies compare? The six studies vary in the proportion of brains collected in relation to their sample populations. The Hisayama study and Vantaa 85+ have autopsied between 70% and 82% (depending on years) and 52% of respondents respectively. These are remarkable autopsy rates in the context of a global decline in autopsies [102]. HAAS' autopsies were discussed with all examined participants at the 1991–93 and 1994–96 examinations and the study has collected 559 brains so far (equal to 15% of the number of participants at the 1991 baseline). CFAS has performed 470 autopsies and another 250 are pledged. This is equal to around 40% of those approached to take part in the donation programme. Factors such as whole body donation, non-notification of death, coordination difficulties or changes of decisions at or before death, all result in erosion of the 'population representativeness' of the autopsy cohort. The possibility that systematic biases relate to the willingness of respondents to countenance brain donation has not often been studied. In one study of healthy elderly people, agreement to brain donation was related only to age and the Cornell Depression Scale score. Older respondents (≥ 85 years of age) were more likely to consent to donation than younger ones [103]. Another study investigating the differences in pre-morbid clinical diagnoses between autopsied and non-autopsied dementia patients, the autopsied individuals differed in age, race, and interval between the last clinical contact and death compared to those who did not [104]. Those patients with early onset of disease (<65) were more likely to consent to autopsy than those whose illness began somewhat later in life. A key motivating factor for agreeing to post-mortem examination is personal knowledge of an AD patient [105]. These findings are reassuring and suggest that even though autopsies are voluntary, they do not seem to be skewed to one segment of the demented population.

HAAS, Vantaa 85+, CFAS and CC75C have all addressed the representativeness of their autopsy group. In HAAS the autopsy sample was similar to the total cohort in terms of sociodemographic terms, cognitive status and late life cholesterol levels. The autopsy rate was approximately 50% among the participants who had been recognised as demented with the autopsy rates being similar for the four clinically diagnosed subtypes of dementia [45,61]. Vantaa 85+ reported slight differences in the age and sex between the autopsied and the non-autopsied subpopulations [27]. CFAS and CC75C used sampling strategies that enriched the autopsy sample to include older and more impaired respondents but other than these characteristics there was no clear indication that the donor sample differed from the general population in basic sociodemographic indicators [20,34].

Even though brain donations may not be biased regarding dementia status, other factors could introduce error into the autopsy process such as lack of notification of the study team because of the place of death: hospital, institution or home. The rapid accessibility to the body and the idea that it is likely that cognitive decline has a difference course in patients who are institutionalised compared to those who remain at home until the end of their life both play a part in the generalisability of the findings. One must assess the impact of only including a small proportion of individuals who die at home in an autopsy study [106]. HAAS clearly reported the place of death of its cohort members whom have undergone autopsy, this being 71% in the hospital. 13.1% at home, 11.2% in a nursing home and 4.6% in a hospice or other place [61] although none of the studies have reported on 'missed' autopsies.

### Case identification, examination and in life diagnosis

Even if the initial cohort is population-based, studies may lose their population representation because of their case identification strategy. This arises if they rely on studying cases that have already come in contact with the health care system. For example, one of the excluded studies, the University of Washington Alzheimer's Disease Patient Registry (ADPR), uses a base population comprising members of a Group Health Cooperative with an attrition rate less than 1% per year. However, cases are found through the primary care physician network, referral from neurologists and mental health services or by monitoring events leading to the entry of demented patients into the health-care system [107]. Dementia cases remaining in the community are therefore under-represented. There is no organised population-based or primary-care effort directed at the early detection of dementia in the USA and in the UK primary care physicians have reported that fewer than 50% have adequate basic and post-qualifying training for identifying dementia [108]. One can fairly assume that many dementia cases go unnoticed in the health care system and that such a design results in a sample with limited population representation.

All the studies reported here have used direct interviewing of the populations identified with standardised assessments (Table 2). Clinical assignment of a dementia diagnosis was undertaken in three of the studies by consensus between expert clinicians. The Cache County study set up diagnostic conferences including geropsychiatric, neurology, neuropsychology and cognitive neuroscience expertise and the final diagnosis was achieved using information from all phases of the study including the autopsy results. HAAS and Vantaa 85+ achieved clinical diagnosis through smaller clinician panels and CC75C used a single psychiatrist with inter-rater reliability tests. CFAS based its findings on a previously validated clinically based computer-assisted taxonomy (AGECAT) administered by trained lay research interviewers [109].

### Neuropathological case confirmation

The comprehensiveness and comparability of the neuropathological investigation varies more in population settings. All more or less closely followed the Consortium to Establish a Registry for Alzheimer's Disease (CERAD) neuropathology protocol [13]. This is in contrast to the broader variation in methods for clinical diagnosis (Table 2). Details of neuropathological investigations are yet not available in the published literature for the Cache County Study. In Vantaa 85+, besides completing the CERAD protocol, the NIA-RIA criteria was also adopted which emphasises topographic staging of neurofibrillary changes in addition to neuritic plaques [110,111], and the investigators routinely sampled additional areas of the brain supplementing the usual techniques with immunocytochemical methods to enhance detection of amyloid and Lewy bodies. Regarding how many neuropathologists carried out the examination: in CFAS, the Hisayama study and HAAS, postmortem investigation was performed by several neuropathologists, while in the Vantaa 85+ study, for one study looking at brain infarctions, the examination was done by one pathologist. No information is given for the Cache County Study. Comparability of assessment has been checked within CFAS and HAAS.

The CERAD protocol gives guidelines for tissue fixation, tissue processing, sectioning and tissue staining. It provides a simple semiquantitative systematic gathering of information on clinical, neuropsychological and neuropathologic aspects reducing subjective interpretation [13,112]. Nonetheless, the assignment of probability of a diagnosis of AD by the CERAD approach is based on concepts of clinicopathological correlation derived from cohort studies of selected cases and 'controls' so that it may represent a biased view of what burden of lesions are associated with a positive diagnosis for dementia. For instance, CERAD uses abundant neuritic plaques as a reference for AD case definition, reinforcing the concept of AD as a disorder of amyloidosis. The relationship between lesion burden and cognitive status, and the interactions between lesions, differs in a population sample from those in selected secondary referral cohorts of demented people [34]. It is increasingly acknowledged that the proportion of normal elderly individuals with a substantial neuropathological burden is not negligible [34,106,113]. The logical aim of neuropathological studies of dementia, if no neuropathological 'gold standard' exists, would be to collect information in an unbiased way to test which diagnostic criteria are most valid and reliable.

The CERAD protocol was developed in the 1980s and is based on a paradigm which heavily relied on the significance of lesions such as neurofibrillary tangles and amyloid plaques as the pathological substrate of cognitive decline. The accumulation of data from many studies over the last 15 years underline the limitations of this approach. The CERAD protocol ignores a number of pathological and biochemical advances which have identified additional targets, or refined the basis for quantifying pathologies, that are likely to have a closer link with cognitive decline or are additional pathologies that are not taken into account. These newer approaches include: biochemical estimations of soluble amyloid peptide load and aberrant tau accumulation in brain regions, synaptic density measurements, glial responses such as astrogliosis and microgliosis, severity of white matter attenuation. Finally CERAD provides no basis for assessing the significance of microvascular pathologies in the causation of cognitive decline.

A significant limitation is imposed through the necessity to adopt a validated pathology protocol with documented inter-laboratory comparability. In CERAD, 83% of raters showed consistency for plaque determination, whereas only 66% showed consistency for tangle determination. Despite this, apart from bias resulting from knowledge of dementia status before death, most of the other types of variation should produce non-systematic bias and therefore underestimate any relationship [11]. Mirra has remarked that 'whether this variation is produced by differences in the phase of the moon or other indeterminate factors remains unclear, but, in my view, standardisation of methodology will be difficult to achieve' [114]. The six studies report few measures taken to assure or measure quality in the neuropathological investigation. Blinding of the neuropathologist to clinical and risk factor data is reported for Vantaa 85+, CC75C and CFAS. Neuropathological inter-rater reliability was only described by CFAS.

### Timing of data collection

Each study up-dated their data in life around every 2 years. In population studies it has been accepted that it is not feasible to have regular clinical follow-up until death for each member of a large study cohort despite the desirability of minimising the time interval between the last examination and death [106]. This interval is critical in determining whether pathological features at autopsy are a reflection of the cognitive state at last interview. Health checks were done at least once a year in the Hisayama study. For Vantaa 85+ the average length of time from the last clinical examination to death was 0.99 y (range 0.05 – 2.35 y), for HAAS, the average was 2.4 y (SD 0.9 y) and for CFAS 1.2y (range of 3 d – 4.2 y; 81% within 2 y). In CC75C the study design and timing of the interview waves suggests the interval between last clinical examination and death is usually less than 3 years unless follow up interview was refused. Details have not been published yet for the Cache County study. Using various informant interviews may help in gaining a truer cognitive picture of the participant if delays between follow-up and death are considered too long. Studies which report the use of informant interviews can be found in (Table 2).

The timing of autopsy and methods of handling after death are also important for molecular studies. Formalin fixing plays a role in any future neuropathological examination and affects the sensitivity of the immunocytochemistry results. This variable depends on the work force available to complete the neuropathological examinations at the time of death and is thus dependent on the level of funding for the study and the relative importance given to such studies in their research and cultural contexts. None of the studies have reported time between death and autopsy although this is usually available in mortuary records.

### Comparing and combining studies

Research based on community samples has the advantage that it allows the calculation of attributable risk, population attributable risk and population excess risk, which can all estimate how important a given factor is in a particular population. Each study has used similar neuropathological standards but this does not necessarily imply that the results are directly comparable. This is illustrated by the example of the classification of 'possible' and 'probable' cases of dementia. HAAS has described mutually exclusive subcategories of dementias: AD as primary cause, Vascular Dementia (VaD) as primary cause, mixed AD/VaD and other types of dementias [63] rather than risking misclassification of mixed cases; whereas in the Vantaa 85+ study, no 'mixed dementia' category is mentioned [27]. Combining of clinical information should be possible if one goes back to specific measures within each study. Unfortunately there is little overlap in the areas investigated amongst the six existing population-based neuropathological studies so that a systematic review of their results is not possible.

### Redefining the field

Despite all the effort put into setting up the studies and gathering evidence, these datasets remain as yet under-tapped resources. So far most population-based studies have used post-mortem information to test or validate clinical diagnoses. Few have taken the opportunity to challenge and re-characterise existing criteria. Until relatively recently clinicopathological studies have concentrated on clinically clear-cut cases of AD but these population-based studies have confirmed that there is considerable overlap in pathologies found in the demented and non-demented [20,34,45]. For example in HAAS, 33% of demented subjects' condition could not be attributed to any of four primary pathogenic processes or to a combination of them (vascular lesions, AD lesion patterns, hippocampal sclerosis and cortical Lewy bodies). The same heterogeneity of lesions was found in CC75C. In CFAS' first 209 autopsies, both cerebrovascular (78%) and Alzheimer type (70%) pathology were common. Dementia was present in 48% of whom 64% had features indicating probable or definite AD. 33% of the non-demented people, however, had equivalent densities of neocortical neuritic plaques. Some degree of neocortical neurofibrillary pathology was found in 61% of demented and 34% of non-demented individuals. Vascular lesions were equally common in both groups, although the proportion with multiple vascular pathology was higher in the demented group (46% vs. 33%). In the same way, Vantaa 85+ findings show that the prevalence of neuropathologically defined AD was 33% whereas the prevalence of clinically diagnosed AD was 16%. Although there was a correlation between clinical AD and neuropathological AD, 55% of individuals with neuropathologic AD were either in the non-demented group or in the Vascular dementia (VaD) or other clinical non-AD groups. On the other hand, 35% of those with clinical AD did not fulfil the neuropathologic criteria for AD used in the study. The Cache County study reported 21% of mixed dementia diagnosis using a consensus between clinical and autopsy findings.

## Conclusion

The aim of this paper was to carry out a systematic review of all existing population-based neuropathological studies of old age dementia to assess the representativeness of their study populations and to review their neuropathological investigation methods. The review considers study design and the range of investigations undertaken. Referral and volunteer cohort studies tend to hold more complete and extensive information and have fewer problems in terms of attrition. Population-based studies on the other hand demand prodigious organisation and effort over a prolonged period and result in a potentially rich source of data to help understand the aetiology and pathogenesis of late life dementia with less bias. The six studies together with their 1,200+ brains directly relate to a base population of near 32,000 people. New work could be indicative such as testing genetic polymorphisms. They have the ability to take the valuable findings from clinical series and volunteer cohorts and estimate how important findings are for the community as they have the only setting which permit an unbiased extrapolation of the results to the general population. With their abundance of recorded clinical and neuropsychological information, these studies could help in the constructive challenge to existing neuropathological criteria and create new hypotheses about the biological substrates of cognitive, functional and behavioural changes with age. These studies are rare globally and can provide unique insights. Individually they are limited in power and scope; together they represent a powerful resource to translate findings from laboratory to populations.

## Authors' contributions

CB developed the concept for the project. JZ formulated the search strategy, determined the protocol for the systematic review and carried out the data extraction. The manuscript was prepared by JZ and CB and reviewed by JZ, CB and PI. All authors read and approved the final manuscript.

## Pre-publication history

The pre-publication history for this paper can be accessed here:



## Supplementary Material

Additional File 1 Selected sample of non-population-based neuropathological studies of old age dementia [7,107,134-140]. Click here for file

Additional File 2 Selected paper titles to illustrate areas of investigation from the five population-based neuropathological studies of old age dementia [15,18-20,25-29,33,34,38,45,46,51,53,55,61,64,78,141,142]. Click here for file

## Figures and Tables

**Table 1:** Key information from the community-based neuropathological studies of old age dementia

Study	Hisayama study	CC75C	Vantaa 85+	CFAS		HAAS	Cache County study
						
				Nottingham Gwynedd Newcastle Cambridgeshire Oxford	Liverpool*		
Start date	1985 (for dementia prevalence)	1985–1987	1991	1991–93	1989–91	1991–1993	1995–1997
Number of subjects eligible	1440 in 1992	2,835	601	16,261	6,028	4,678	5,677
Years for re-examination	yearly	1988–90 1991–92 1994–95 1998–99 2002–2003	1994 1996 1999	1992 1995 1998 2000 2002	1991–93 1997–98 1999 2002	1994–1996 1997–1999 2000–2001 2002–2003 2004- in progress	1998–2000 2004- in progress
Number of subjects followed initially (% of those eligible)	1436 (99.7%) in 1992	2609 (92%)	553 (92%)	13009 (80%)	5222 (87%)	3734 (79.8%)	5092 (89.7%)
Age at start of study, mean (range) in years	Men 73 (65–92) Women 74 (65–98)	81 (75–106)	89 (85–90+)	75 (65–105)^	79 (65–108)	78 (71–93)	74 (65–90+)
% men at start of study	40	34	21	40	47	100	42
Number of brains collected	176 (1985–1992) + 105 (1998–2001)	225 (as of mid 2004)	305 (as of 2002)	370	103 (as of mid 2004)	559 (as of 2004)	65 (+5 whom have not completed clinical assessment) (as of 2002)

* different interviewing strategy than the other 5 centres

^ stratified to have equal numbers above and below 75

**Table 2:** Clinical and neuropathological standards and tests followed by the selected studies

Studies			Hisayama Study	CC75C	Vantaa 85+	CFAS	HAAS	Cache County study
Dementia clinical diagnosis criteria	Screening tools and general measures for cognitive decline	MMSE	X	X	X	X		X
		3MS						X
		IQCODE					X	X
		HAS				*		
		SPMSQ			X			
		MHIS				X		X
		Cummings-Benson					X	
		Blessed dementia scale		*		X		
		Neuropsych. Inventory						X
		Dementia Questionnaire		*				X
		CASI						
		CAMDEX		*		X		
		AGECAT				X		
		CAMCOG		*		X	X	
		GMS				X		
		CDR			X	X	X	X
		DSR						X
		DSM-III-R	X	*	X	X	X	X
	Dementia type diagnosis criteria	NINCDS-ADRDA	X		X		X	X
		IWG-NINCDS-AIREN	X		X			
		ADDTC					X	
		Hasegawa's Dementia Scale	X					
Neuropat hological investigation standards		NIA-RI			X			
		CERAD	X	X	X	X	X	X
Blood sample collected				* in some	X	* in some	X	X
Neuroimaging			X in some		* postmortem MRI on 145 brains		X	X
Depression diagnosis criteria	Hamilton Depression Scale			X				
	Clinical Interview for Depression			X				
	Nottingham Life Satisfaction Index			X				
	Zung Depression Status Inventory		X		X			
Activities of Daily Living	ADL		X	X	X	X		
	IADLS			*	X	*		
	Modified Townsend ALD Scale					X		
Other							Neurologic examination Hearing and vision tests	Videos East Boston Memory Tests

X Data routinely collected with some mention in published literature

* Data known to have been collected although not referred to yet in the literature

3MS – modified Mine Mental State Examination [116]

ADDTC – California Alzheimer Disease Diagnostic and Treatment Centers (used for clinical diagnosis of Vascular Dementia) [117]

ADL – Activities of Daily Living [118]

AGECAT – Automated Geriatric Examination for Computer-Assisted Taxonomy [119]

CAMCOG – Cambridge Cognition Examination [120]

CAMDEX – Cambridge Examination for Mental Disorders of the Elderly [121]

CASI – Cognitive Abilities Screening Instrument [122]

CDR – Clinical Dementia Rating [123]

DSR – Dementia Severity Rating Scale [124]

GSM – Geriatric Mental State [125]

HAS – History and Aetiology Schedule (informant interview) [109]

IADLS – Instrumental Activities of Daily Living Scale [126]

IQCODE – Informant Questionnaire on Cognitive Decline in the Elderly [127]

IWG-NINCDS-AIREN – International Work Group of the National Institute of Neurological and Communicative Disorders and Stroke and the Association Internationale pour la Recherche et l'Enseignement en Neuroscience (used for the clinical diagnosis of Vascular Dementia) [128]

MMSE – Mini Mental State Examination [129]

MHIS – Modified Hatchinski Ischaemic Score [130, 131]

NINCDS-ADRDA – National Institute of Neurological and Communicative Disorders and Stroke and the Alzheimer's Disease and Related Disorders Association [132]

SPMSQ – Short Portable Mental Status Questionaire [133]
