# Supplementary material for: Population-based neuropathological studies of dementia: design, methods and areas of investigation – a systematic review
Source: BMC Neurol. 2006 Jan 9;6:2. doi: 10.1186/1471-2377-6-2 (PMC1397861; doi:10.1186/1471-2377-6-2)
Supplement: Additional File 1 — Selected sample of non-population-based neuropathological studies of old age dementia [7,107,134-140]. [file 1471-2377-6-2-S1.doc]

Selected sample of non-population-based neuropathological studies of old age dementia [7, 107, 134-140].

| Study name | Characteristics |
| --- | --- |
| Sydney Older Persons Study [7] | Random sample of 647 community-dwelling only men and women aged 75 or over and residing in the Central Sydney Area Health Service. |
| “Shimane Prefecture” study [134] | Subjects were nursing home residents of the Shimane Prefecture, Japan, who had consented to bequeath their bodies for medical science after nursing home admission. |
| Rush Religious Order Study [135] | Group of 851 older Catholic nuns, priests and brothers without dementia at baseline who underwent detailed annual clinical evaluations and brain autopsy at death. |
| Nun study [136] | Respondents are members of the School Sisters of Notre Dame religious congregation who all agreed to brain donation at death. |
| Bronx Aging Study [137]  Oregon Brain Aging Study [138]  Baltimore Longitudinal Study of Aging [139] | Volunteers were recruited via the press and community centres. |
| Vienna Longitudinal Study on Dementia [140] | Respondents were solely recruited from two major geriatric centres in Austria rather than from the general population. |
| University of Washington Alzheimer’s Disease Patient Registry (ADPR) [107] | ADPR solely evaluates persons with dementia who came to medical attention through an on-going surveillance system. There was no screening of the population. |
