# Supplementary material for: Population-based neuropathological studies of dementia: design, methods and areas of investigation – a systematic review
Source: BMC Neurol. 2006 Jan 9;6:2. doi: 10.1186/1471-2377-6-2 (PMC1397861; doi:10.1186/1471-2377-6-2)
Supplement: Additional File 2 — Selected paper titles to illustrate areas of investigation from the five population-based neuropathological studies of old age dementia [15,18-20,25-29,33,34,38,45,46,51,53,55,61,64,78,141,142]. [file 1471-2377-6-2-S2.doc]

| Area of investigation | Study | Paper title and objectives | Findings |
| --- | --- | --- | --- |
| Clinicopathological correlation and use of postmortem-confirmed diagnosis | Hisayama Study | Incidence and risk factors of dementia in a defined elderly Japanese population [15].  Objective: To define the incidence and risk factors of dementia. | VaD is more common than AD.  Prevalence of VaD decreased for men, while AD remained unchanged in both sexes during a 7-year follow-up period (1985-1992).  Decreased prevalence of VaD seems to be due to decreased incidence of stroke in recent years.  Risk factors for VaD were age, hypertension, previous stroke, and alcohol consumption, while age was only a significant risk factor for AD. |
|  |  | Age-associated prevalence and risk factors of Lewy body pathology in a general population: the Hisayama study [18].  Objective: To investigate the prevalence of LB pathology in a community-based population and to evaluate the relationship between LB and ATD pathology. | LB pathology was present in 23 (22.5%) of 102 autopsies.  LB pathology was present in12 (41.4%) of the demented subjects.  The LB score was not significantly different between DLB cases and non-demented subjects with LB pathology.  Braak stages were significantly different between the two groups.  Prevalence of LB pathology constantly increased with age. |
|  | CC75C | Neuropathological findings in the very old –results from the first 101 brains of a population-based longitudinal study of dementing disorders [20].  Objective: To report neuropathological findings in demented and non-demented participants. | Heterogeneity of lesions in very old populations.  Considerable overlap in the pathologies found in the demented and non-demented.  White matter (ischemic) pallor and amyloid angiopathy, as well as neuritic plaques, neurofibrillary tangles and LB formation are all lesions that increase the likelihood of dementia. |
|  | Vantaa 85+ | Prevalence of Alzheimer’s disease in very elderly people [27]  Objective: To study the point prevalence of neuropathologically defined AD in a population of people at least 85 years of age, stratified according to their APOE genotype. | The prevalence of neuropathologically defined AD was 33%.  Prevalence of clinically diagnosed AD was 16%.  Highly significant (p < 0.001) association between the APOE epsilon4 allele and AD: 63% of APOE epsilon4 carriers and 20% of non-carriers had neuropathologic AD. The respective figures in subjects aged 90 years or more were 71 and 22%.  The prevalence of neuropathologically defined AD is higher than that reported in most previous studies based on clinical diagnosis. |
|  | CFAS | Neuropathological correlates of late-onset dementia in a multicentre, community-based population in England and Wales [34].  Objective: To test the assumption that dementing disorders as defined by current diagnostic protocols underlie this syndrome in the community at large. | Cerebrovascular (78%) and Alzheimer-type (70%) pathology were common.  Dementia was present in 100 (48%), of whom 64% had features indicating probable or definite AD.  33% of the 109 non-demented people had equivalent densities of neocortical neuritic plaques.  Some degree of neocortical neurofibrillary pathology was found in 61% of demented and 34% of non-demented individuals.  Vascular lesions were equally common in both groups, although the proportion with multiple vascular pathology was higher in the demented group (46% vs. 33%). |
|  |  | Vascular pathologies and cognition in a population-based cohort of elderly people [141].  Objective: To compare vascular pathologies and cognition in a population-based cohort of elderly people. | White matter lesions are common (94% overall frequency) and are an independent risk factor for dementia using multivariable analysis. |
|  | HAAS | Cerebrovascular pathology and dementia in autopsied HAAS respondents [45].  Objective: To assess the prevalence at death of four primary neuropathologic processes using specific microscopic lesions as indicators. | An algorithm was developed to assign each decedent to one of six subsets, corresponding to pathologic dominance by microvascular lesions (14% of decedents), Alzheimer lesions (12%), hippocampal sclerosis (5%), cortical LB (5%), codominance by two or more primary processes (9%), or without a dominant pathologic process recognized (55%).  The proportions of men in each subset identified as demented were (in the same order) 57%, 53%, 79%, 57%, 76%, and 25%.  Importance of microvascular lesions as a likely explanation for dementia was nearly equal to that of Alzheimer lesions. |
|  |  | Cerebral amyloid angiopathy and cognitive function [61].  Objective: To investigate the relationship between cerebral amyloid angiopathy (CAA), dementia, and cognitive function in an autopsy sample of 211 Japanese-American men. | 44.1% of subjects had CAA in at least one neocortical area.  Presence of CAA was associated with higher mean NFT and NP counts and having at least one APOE-epsilon4 allele.  The interaction between CAA and AD on the adjusted mean CASI score was significant: compared with non-demented men without CAA, the CASI score was 16.6% lower in men with AD and no CAA and 45.9% lower in men with AD plus CAA. |
|  | Cache County study | Incidence of AD may decline in the early 90s for men, later for women [64].  Objective: To characterize the incidence of AD among the elderly population of Cache County to explore sex differences and to examine whether AD incidence plateaus or declines in extreme old age. | Incidence of both dementia and AD increased almost exponentially until ages 85 to 90, but appeared to decline after age 93 for men and 97 for women.  A statistical interaction between age and the presence of two APOE-epsilon 4 alleles indicated acceleration in onset of AD with this genotype; the interaction of age and one epsilon 4 suggested more modest acceleration.  A statistical interaction of sex and age indicated greater incidence of AD in women than in men after age 85. |
| Assessing standards and staging models | Hisayama study | * |  |
|  | CC75C | The relationship between clinical dementia and neuropathological staging (Braak) in a very elderly community sample [26].  Objective: To assess the relationship between clinical dementia and neuropathological (Braak) staging. | Demented cases had significantly more areas involved and more advanced neuropathological stages.  Cases with Braak stages 1-3 tended to be non-demented, and cases with stages 4-6 tended to be demented.  There was a considerable degree of overlap and no clear-cut threshold could be established. |
|  |  | Examination of the validity of the hierarchical model of neuropathological staging in normal aging and AD [25].  Objective: To assess the relationship between clinical dementia and neuropathological (Braak) staging. | There was a significant correlation between the overall extent of neurofibrillary pathology and the number of regions affected.  There were frequent order violations in the proposed hierarchy: 19 instances (45%) involving entorhinal and transentorhinal cortices, and 16 instances (38%) involving CA1 of hippocampus and entorhinal cortex. Only 6 out of 42 cases conformed in all regions to the expected hierarchy.  90% of the cases had 2 order violations or less, supporting the approximate validity of the hierarchy. |
|  | Vantaa 85+ | * |  |
|  | CFAS | * |  |
|  | HAAS | Accuracy of clinical criteria for AD in the HAAS, a population-based study [46].  Objective: To determine diagnostic accuracy for AD in a population-based study of Japanese-American men. | Using established neuropathologic criteria, 25% (5/20) of clinical AD cases had enough NP to meet definite AD criteria, whereas 65% (13/20) had sufficient NP to meet neuropathologic definite or probable AD criteria.  Among nine AD cases with moderately severe dementia, only two (22%) had NP densities great enough to meet definite neuropathologic criteria, whereas seven (78%) met neuropathologic criteria for probable AD. |
|  | Cache County study | * |  |
| Association between neuropathologies and risk factors | Hisayama study | Incidence and risk factors of vascular dementia and Alzheimer’s disease in a defined elderly Japanese population [19].  Objective: To determine the type-specific incidence of dementia and its risk factors in the general Japanese population. | Incidence of VaD and AD increased with age for both sexes.  Age-adjusted total incidence (per 1,000 person-years) of dementia was 19.3 for men and 20.9 for women.  Corresponding rates for VaD were 12.2 for men and 9.0 for women, and for AD, 5.1 for men and 10.9 for women (per 1,000 person-years).  Among the VaD subjects, the most frequent type of stroke was multiple lacunar infarcts (42%) but half these subjects lacked a stroke episode in their histories.  Multivariate analysis showed that age, prior stroke episodes, systolic blood pressure, and alcohol consumption were significant independent risk factors for the occurrence of VaD.  Age and a low score on Hasegawa's dementia scale were significant risk factors for AD, and physical activity was a significant preventive factor for AD. |
|  | CC75C | * |  |
|  | Vantaa 85+ | * |  |
|  | CFAS | * |  |
|  | HAAS | Midlife blood pressure and neuritic plaques, neurofibrillary tangles and brain weight at death [55].  Objective: To investigate the relationship of BP level in midlife to development of NFT, NP and low brain weight at autopsy. | Elevated systolic BP (> or =160 mm Hg) in midlife was associated with low brain weight and greater numbers of NP in both neocortex and hippocampus.  Diastolic BP elevation (> or =95 mm Hg) was associated with greater numbers of NFT in hippocampus.  There is a direct relationship with brain atrophy, NP and NFT. |
|  |  | Cholesterol and neuropatholgic markers [51].  Objective: To examine the association of plasma cholesterol (total and high-density [HDL] and low-density lipoprotein) levels with NP and NFT in a population-based autopsy series of 218 Japanese American men. | After adjusting for age at death, education, APOE allele, dementia, neuropathologic infarction, and blood pressure, a strong linear association was found for increasing late-life HDL cholesterol (HDL-C) levels and an increasing number of neocortical NP (5th versus 1st quintile: count ratio [95% CI] 2.30 [1.05 to 5.06]) and hippocampal (2.63 [1.25 to 5.50]) and neocortical (4.20 [1.73 to 10.16]) NFT.  Trends were similar for the midlife HDL-C levels. |
|  | Cache County study | * |  |
| Genetic investigation | Hisayama Study | * |  |
|  | CC75C | Apolipoprotein E genotype in the prediction of cognitive decline and dementia in a prospectively studies elderly population [142].  Objective: To assess the influence of Apoe alleles on both dementia and cognitive decline in the elderly, the ApoE genotype was determined in 150 individuals over the age of 75 years. | Homozygosity for the epsilon 4 allele was rare.  Of the 2 homozygotes, 1 was severely demented but the other did not receive a clinical diagnosis of dementia. The latter individual demonstrated marked cognitive decline over a 28-month period.  There was a consistent association between the presence of an epsilon 4 allele and both the clinical diagnosis of dementia and cognitive decline. |
|  | Vantaa 85+ | Association of lipoprotein lipase Ser447Ter polymorphism with brain infraction [29].  Objective: To test for an association between brain infarction and two common polymorphisms of the LPL gene, Ser447Ter and Asn291 Ser. | LPL Ter447 variant was negatively associated with neuropathologically verified brain infarcts (P = 0.006), and even more strongly with small brain infarcts (P = 0.004).  Ter447 variant was associated with higher serum HDL cholesterol (P = 0.004) and lower triglyceride levels (P= 0.003), and was negatively associated with pathologically verified severe coronary artery disease (P=0.001).  Asn291Ser polymorphism was not significantly associated with brain infarction. Ter447 variant of LPL is associated with decreased risk of brain infarction and coronary artery disease in our very elderly population. |
|  |  | Apoe3-haplotype modulates Alzheimer β-amyloid deposition in the brain [33].  Objective: To test whether the haplotype background of the most common ApoE allele, epsilon3, influences brain amyloid deposition or the risk of neuropathologically verified AD in a population-based sample of elderly Finns. | Epsilon3-haplotypes containing the promoter allele -219T were associated with reduced amyloid deposition and reduced risk of neuropathologically verified AD as compared to epsilon3-haplotypes containing -219G.  The functional polymorphism(s) responsible for the haplotypic difference remains to be identified. |
|  |  | Genetic association of a2-macroglobulin with AD in a Finnish elderly population [28].  Objective: To investigate the relation between two polymorphisms in the a2-macroglobulin gene with the presence of the disease and also with the extent of pathological changes in the cerebral cortex. | A/A genotype in exon 24 of the alpha2-macroglobulin gene was associated with neuropathologically defined diagnosis of AD according to the CERAD criteria and with an increase in the neocortical beta-amyloid protein load.  The effect of this association was stronger in the apolipoprotein E epsilon4-negative group. |
|  | CFAS | Alzheimer disease is not associated with polymorphisms in the angiotensinogen and renin genes –part CFAS sample [38].  Objective: To study the presence of an MboI site in an RFLP in the renin gene and polymorphisms at codon 235 (M235T) of the angiotensinogen gene in autopsy-confirmed AD cases and matched controls from the U.K.  For other similar papers see: www-cfas.medschl.cam.ac.uk | While no association was detected with the renin polymorphism, a weak deleterious effect was observed in cases homozygous for the angiotensinogen Thr allele. |
|  | HAAS | Type 2 diabetes, APOE gene and the risk for dementia and related pathologies [53].  Objective: To evaluate the association of diabetes alone or combined with the apolipoprotein E (APOE) gene with incident dementia and neuropathological outcomes. | Diabetes was associated with total dementia (RR 1.5 [95% CI 1.01-2.2]), AD (1.8 [1.1-2.9]), and VaD (2.3 [1.1-5.0]).  Individuals with both type 2 diabetes and the APOE epsilon4 allele had an RR of 5.5 (CI 2.2-13.7) for AD compared with those with neither risk factor.  Participants with type 2 diabetes and the epsilon4 allele had a higher number of hippocampal neuritic plaques (IRR 3.0 [CI 1.2-7.3]) and neurofibrillary tangles in the cortex (IRR 3.5 [1.6-7.5]) and hippocampus (IRR 2.5 [1.5-3.7]), and they had a higher risk of cerebral amyloid angiopathy (RR 6.6, 1.5-29.6). |
|  | Cache County study | Apoe-e4 count predicts age when prevalence of AD increases, then declines [78].  Objective: To examine the prevalence of AD and other dementias in relation to age, education, sex, and genotype at APOE. Recent studies suggest age heterogeneity in the risk of AD associated with the APOE genotype and a possible interaction between APOE-epsilon4 and female sex as risk factors. | The adjusted prevalence estimate for AD was 6.5% and for all dementias 9.6%.  After age 90, the adjusted prevalence estimate for AD was 28% and for all dementias 38%.  Regression models showed strong variation in AD prevalence with age, sex, education, and number of epsilon4 alleles (effect of epsilon2 not significant).  Models were improved by a term for age-squared (negative coefficient) and by separate terms for interaction of age with presence of one or two epsilon4 alleles.  An association of AD with female sex was ascribable entirely to individuals with epsilon4.  In participants with no epsilon4 alleles, the age-specific prevalence of AD reached a maximum and then declined after age 95. In epsilon4 heterozygotes a similar maximum was noted earlier at age 87, in homozygotes at age 73. |

* No papers published on this subject as of early 2005.

AD = Alzheimer’s disease; BP = blood pressure; CAA = cerebral amyloid angiopathy; CAMDEX = Cambridge Examination for Mental Disorders of the Elderly; CASI = Cognitive Abilities Screening Instruments; CERAD = Consortium to Establish a Registry for Alzheimer's Disease; DLB = Dementia with Lewy bodies; LB = Lewy bodies; NFT = neurofibrillary tangles; NP = neuritic plaques; VaD = Vascular dementia.
